# An amino acid mixture, enriched with Krebs cycle intermediates, enhances extracellular matrix gene expression in cultured human fibroblasts

**DOI:** 10.1007/s00726-023-03340-y

**Published:** 2023-09-28

**Authors:** Maurizio Ragni, Luca Canciani, Letizia Spataro, Chiara Ruocco, Alessandra Valerio, Enzo Nisoli

**Affiliations:** 1 Center for Study and Research On Obesity, Department of Medical Biotechnology and Translational Medicine, University of Milan, Via Vanvitelli, 32, 20129 Milan, Italy; 2 Department of Molecular and Translational Medicine, University of Brescia, Viale Europa, 11, 25123 Brescia, Italy

**Keywords:** Amino acids, Extracellular matrix, Aging, Oxidative stress

## Abstract

In the human body, the skin is one of the organs most affected by the aging process. Nutritional approaches aimed to counteract the age-induced decline of extracellular matrix (ECM) deposition could be a valuable tool to decrease the degenerative processes underlying skin aging. Here, we investigated the ability of a six-amino acid plus hyaluronic acid (6AAH) formulation enriched with tricarboxylic acid (TCA) intermediates to stimulate ECM gene expression. To this aim, human BJ fibroblasts were treated with 6AAH alone or plus succinate or malate alone or succinate plus malate (6AAHSM), and mRNA levels of several ECM markers were evaluated. 6AAHSM increased the expression of all the ECM markers significantly above 6AAH alone or plus only succinate or malate. Furthermore, in an in vitro oxidative damage model, 6AAHSM blunted the hydrogen peroxide-induced decline in ECM gene expression. Our data suggest that feeding cells with 6AAH enriched with TCAs could efficiently be employed as a non-pharmacological approach for counteracting skin aging.

## Introduction

Skin aging is characterized by sequential and cumulative alterations in its structure and function, mainly ascribed to a decrease in the production of the extracellular matrix (ECM) component (Shin et al. 2019; Sparavigna 2020). The decline in ECM function causes the loss of skin elasticity and resiliency and the occurrence of wrinkles, which is a conventional feature of skin aging (Black et al. 2008; Birch 2018). Fibroblasts produce ECM mainly composed by proteins and glycosaminoglycans, of which the primary constituent is hyaluronic acid (HA). HA is the key molecule involved in skin hydration since it possesses a high and unique capacity to bind and retain water molecules; as such, a decrease of dermal HA content and activity during aging, resulting from both decreased synthesis or increased degradation by tissue hyaluronidases (HAse), leads to dryness and loss of skin moisture (Papakonstantinou et al. 2012; Scarano et al. 2021).

The protein component of ECM includes fibrous proteins such as collagen, elastin, fibronectin, and laminin, characterized by well-defined amino acid (AA) composition. Elastin (ELN) is the major protein responsible for skin elasticity and comprises about 2% of the total derma proteins (Theocharis et al. 2016). ELN is synthesized through its soluble isoform tropoelastin, formed by alternate hydrophobic and hydrophilic cross-linking domains. The formers are enriched in non-polar AAs such as glycine, valine, proline, and alanine. They are arranged as repeated sequences of three-nine AAs, while the hydrophilic domains are enriched in alanine and lysine. Fibronectin (Fbn) has a crucial role in cell attachment, movement, and adhesion instead; as such, Fbn displays several binding motifs in its protein structure, characterized by definite AA sequences such as Arg-Gly-Asp, Arg-Gly-Asp-Ser, Leu-Asp-Val, and Arg-Glu-Asp-Val, which mediate the cell attachment function of Fbn (Rosso et al. 2004). Collagen (Col) is the most abundant protein in mammals and represents the main extracellular matrix (ECM) component, up to 75% in the dermis. Col has a triple-helix structure that derives from the molecular constraint conferred by a strict sequence of AAs, with arranged repeats of Glycine-X–Y, where glycine stabilizes the triple helix and *X* and *Y* are frequently proline or hydroxyproline; therefore, similar to ELN and Fbn, Col displays a precise AA composition with a high percentage of glycine, hydroxyproline, proline, and alanine. Collagen has 28 family members, numbered from I to XXVIII; however, different isoforms exist within the same collagen type, and alternative promoter usage and other proteolytic cleavage give rise to different levels of diversity inside the family of the Col proteins (Vuorio and De Crombrugghe 1990; Langton et al. 2010; Chu 2011; Dalton and Lemmon 2021).

Besides the decline of structural ECM components, excessive reactive oxygen species (ROS) production is another critical factor in skin aging. According to Harman’s Free Radical Theory of aging, this process is mainly ascribed to a decline in mitochondrial function, which leads to an increase in ROS generation as a by-products of aerobic respiration; consequently, oxidative stress is the leading driver of the well-known phenomenon of premature skin aging by UV, which causes mitochondrial DNA (mtDNA) lesions, dysregulation of oxidative phosphorylation (OXPHOS) and an increase in ROS production (Ziada et al. 2020).

Currently, interventions to delay skin aging are based on preventive approaches such as avoiding excessive sun exposure and/or anti-UV sunscreen use (Mohiuddin 2019). Other therapeutic strategies involve supplementation with natural compounds, such as polyphenols, plant extracts, or HA, to blunt the age-associated dryness of the skin. Accordingly, the definite amino acidic composition of the two major proteins of ECM also provides the rationale for stimulating their synthesis by supplementing their key AA constituents. We have, in fact, previously shown that the treatment of fibroblasts with a specific six AAs (6AA) mixture increases the expression of the ECM components. Furthermore, 6AA induced the expression of antioxidant genes (Tedesco et al. 2022), thus underscoring the effectiveness of AA treatment also as an anti-ROS tool.

Remarkably, both de novo protein synthesis as well as the remodeling and renewal of ECM components, which underlie and support its capacity to sustain mechanotransductive and tensive properties, have a high energy cost, which requires increases in the cell’s mitochondrial ATP output (Romani et al. 2021). Therefore, stimulating mitochondrial activity could help to promote ECM deposition in fibroblasts and, by improving aerobic respiration of the skin, also counteract oxidative stress-induced skin aging. To this aim, a valuable tool could be the supplementation of mitochondrial TCA cycle substrates (TCAs), which has a widespread diffusion to boost oxidative metabolism and increase ATP output or exercise/fatigue resistance in athletes.

On these bases, we have therefore investigated the effects of a 6AA mixture enriched in HA (6AAH) combined with the TCAs succinate, malate, or succinate/malate on the expression of ECM genes in human fibroblasts in both basal conditions and in response to an oxidative stress (hydrogen peroxide) challenge.

## Materials and methods

### Cell cultures and treatment

Human skin fibroblasts BJ (ATCC^®^CRL‐2522™) were cultured (4 × 10^4^ cells) in standard conditions in EMEM medium (Sigma‐Aldrich), supplemented with 10% Foetal Bovine Serum (FBS) (Sigma‐Aldrich) and antibiotics (Sigma‐Aldrich). Cell cultures were incubated at 37 °C, under 95% humidity and 5% CO_2_. Cells were treated with 0.1% (W/V) of the mixtures (composition is reported in Table 1) ± 5 mM each malic and succinic acid (Professional Dietetics S.p.A, Milan-Italy) for 3 days. Untreated cells were plated as controls. Every 24 h, media were replaced with fresh media or amino acid mixtures in both control and treatment flasks. Oxidative stress challenge with Hydrogen peroxide (H_2_O_2_) (500 µM for 2 h) (Sigma‐Aldrich) was performed on cells pre-treated with culture medium only or with the selected mixtures (see Fig. 3) for 24 h; H_2_O_2_ concentration was assessed by performing a preliminary toxicity assay on BJ cells treated with 100-200-500-750-1000 µM H_2_O_2_ for 2 h. Cell viability was measured by cell counting, and 500 µM was then chosen as a sub-lethal dose. At the end of the experimental treatments, cells were collected for mRNA extraction.

**Table 1:** Composition of the 6AAH mixture

Component	% of total
Hyaluronic acid	30
Glycine	30
l-Proline	22.7
l-Leucine	4.2
l-Lysine	3.3
l-Valine	16.8
l-Alanine	22.8

### RNA extraction and quantitative RT-PCR

For the analysis of mRNA levels, 1 µg of total RNA, isolated using the RNeasy kit (Qiagen), was reverse transcribed using iScript cDNA Synthesis Kit (Bio-Rad Laboratories, Segrate, Italy). Triplicate PCR reactions were performed on an iCycler iQ Real-Time PCR Detection System (Bio-Rad Laboratories). Relative gene expression was calculated by a comparative method (2^−ΔΔCt^) using GAPDH as a housekeeping gene. Primer sequences were designed using Beacon Designer 2.6 software (Premier Biosoft International, Palo Alto, CA, USA). Sequences of the specific human primers used are listed in Table 2.

**Table 2:** Primers for qRT-PCR

Gene	Primer Sense (5’–3’)	Primer Antisense (5’–3’)	PCR Product (bp)	*T*_*a*_ (°C)
*Col1a1*	TGATGGTGCTACTGGTGCTG	CCTCGCTTTCCTTCCTCTCC	437	60
*Col4a1*	CTACGTGCAAGGCAATGAACG	GCAGAACAGGAAGGGCATTGT	93	60
*Eln*	TTCCCCGCAGTTACCTTTCC	ACGTTCCCAGGCTTCACTCC	175	60
*Fbn*	GTGGTGTGGTCTACTCTGTGG	TCTGGTCGGCATCATAGTTCTG	434	60
*GAPDH*	GGCTGAGAACGGGAAGCTTGT	CCAGCATCGCCCCACTTGAT	90	60

### Protein extraction and western blotting

Total proteins were extracted with M-PER Mammalian Protein Extraction Reagent (Pierce; ThermoScientific). Protein content was determined with bicinchoninic acid (BCA) protein assays (Pierce); 30–40 µg of proteins were run on 4–20% gradient TGX sodium dodecyl sulfate–polyacrylamide gel electrophoresis (SDS-PAGE) gels (BioRad). Gels were transferred to PVDF, blocked with 5% non-fat dry milk, and incubated with anti-Fbn1 (1:1000, GeneTex, Cat# GTX112794) or anti-Vinculin (1:1000 Cat# V9131, Sigma-Aldrich, Milan, Italy).

## Results

### Addition of TCAs to the 6-amino acids mixture significantly enhances the expression of ECM genes more than 6AA alone

**Fig. 1:**
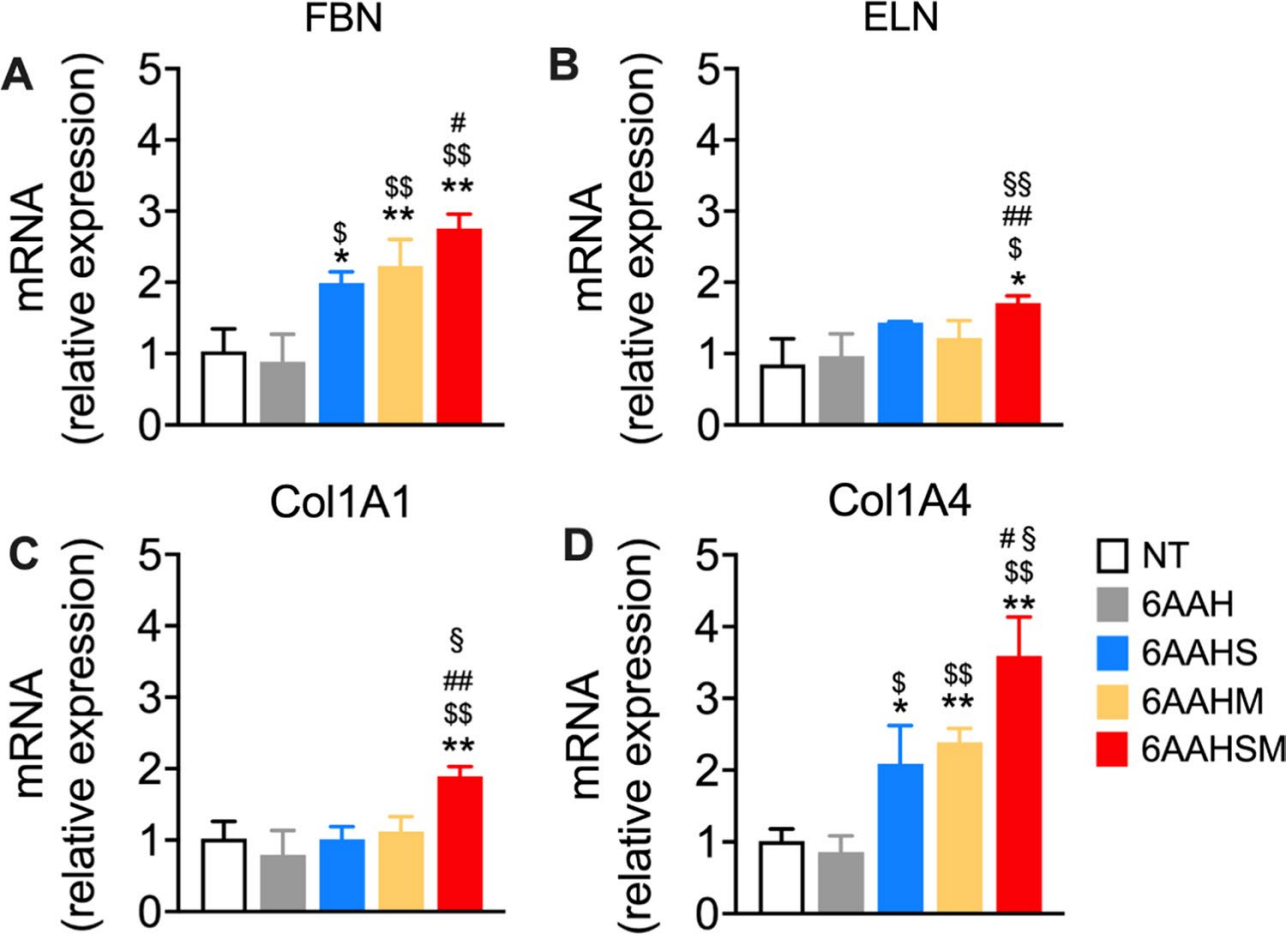
TCAs enhance mRNA expression of ECM markers. Quantitative RT-PCR analysis of ECM genes expression in BJ fibroblasts. Data are Mean ± SEM of triplicate samples of three experimental replicates. NT: untreated controls, 6AAH: six-aminoacid mixture plus hyaluronic acid, 6AAHS: six-aminoacid mixture plus hyaluronic acid and succinate, 6AAHM: six-aminoacid mixture plus hyaluronic acid, and malate, 6AAHSM six-aminoacid mixture plus hyaluronic acid, succinate and malate. ^*^P < 0.05 and ^**^P < 0.01, vs. NT, ^$^*P* < 0.05 and ^$$^*P* < 0.01 vs. 6AAH, ^#^*P* < 0.05 and ^##^*P* < 0.01 vs. 6AAHS, ^§^*P* < 0.05 and ^§§^*P* < 0.01 vs. 6AAHM. One-way ANOVA followed by Tukey’s post hoc test

We have recently reported that the 6AA mixture (*i.e*., glycine, proline, leucine, lysine, valine, and alanine) has been shown to increase ECM gene expression (Tedesco et al. 2022). To enhance its ECM-stimulating effects on cultured fibroblasts, we added HA to the 6AA mixture. We tested if the extra addition of succinic acid (6AAHS), malic acid (6AAHM), or succinic + malic (6AAHSM) to the 6AAH mixture would increase the expression of the ECM markers Fbn, Eln1, and the two collagen isoforms col1a1 and col4a1, concerning only 6AAH, 6AAHM or 6AAHS. Treatment with HA, succinate, malate, or succinate plus malate alone did not affect the expression of any of the ECM genes (data not shown). However, although 6AAH only (at a final concentration of 0.1% w/v) did not change Fbn expression, as compared to control cells (NT) (Fig. 1), the addition of succinate to the 6AAH mixture significantly increased the expression of Fbn mRNA levels concerning both NT and 6AAH (+ 93% and + 124%, respectively). Adding malate to 6AAH also enhanced Fbn expression to 6AAH (+ 152%) and controls (+ 116%). Similarly, the combination of 6AAH with malate plus succinate (6AAHSM) significantly increased Fbn expression above controls (+ 167%) and 6AAH (+ 211%); however, 6AAHSM also induced Fbn expression at a higher magnitude (+ 38%) as compared to 6AAHS alone. When investigating Eln1 expression, only 6AAHSM increased its expression significantly above controls (+ 101%) and the other mixtures, which were almost ineffective. Similarly, the expression of both Col1A1 and Col4A1 was significantly increased by 6AAHSM; in particular, Col1A1 was significantly increased only by 6AAHSM (+ 85% vs. NT), while both 6AAHS and 6AAHM induced significant Col1A4 expression (+ 106% and + 136% *vs.* NT, respectively); however, 6AAHSM treatment increased Col1A4 vs NT at a superior extent to 6AAHS and 6AAHM (+ 255%, + 72% and + 50% vs. NT, 6AAHS, and 6AAHM, respectively).

**Fig. 2:**
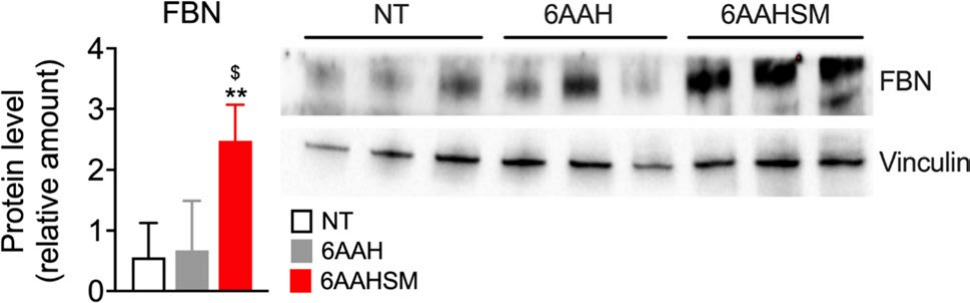
6AAHSM induces Fbn protein expression. Western blot analysis (right panel) of Fbn protein in triplicate BJ samples. Vinculin was used as a loading control. Left panel: quantification of immunoblot data. NT: untreated controls, 6AAH: six-aminoacid mixture plus hyaluronic acid, 6AAHSM: six-aminoacid mixture plus hyaluronic acid, succinate, and malate. ^**^P < 0.01, vs. NT and ^$^*P* < 0.05 vs. 6AAH. One-way ANOVA followed by Tukey’s post hoc test

Fbn has been shown to have a crucial role also in regulating and promoting the deposition of several other ECM components, including collagens (Sottile et al. 2007; Dalton and Lemmon 2021); therefore, we evaluated Fbn protein expression in BJ fibroblasts incubated with culture media, 6AAH or 6AAHSM, which maximally increased Fbn mRNA over other mixtures (Fig. 1). As shown in Fig. 2, 6AAHSM significantly increased Fbn protein expression to untreated or 6AAH-treated BJ fibroblasts, thus further validating mRNA expression data and confirming that 6AAHSM can regulate the deposition of ECM components; moreover, combination of 6AAH mixture with succinate plus malate has a superior ability in inducing ECM gene expression as compared to 6AAH or succinate or malate alone.

### The 6AA mixture plus succinate and malate restores the hydrogen peroxide-induced decline of ECM

**Fig. 3:**
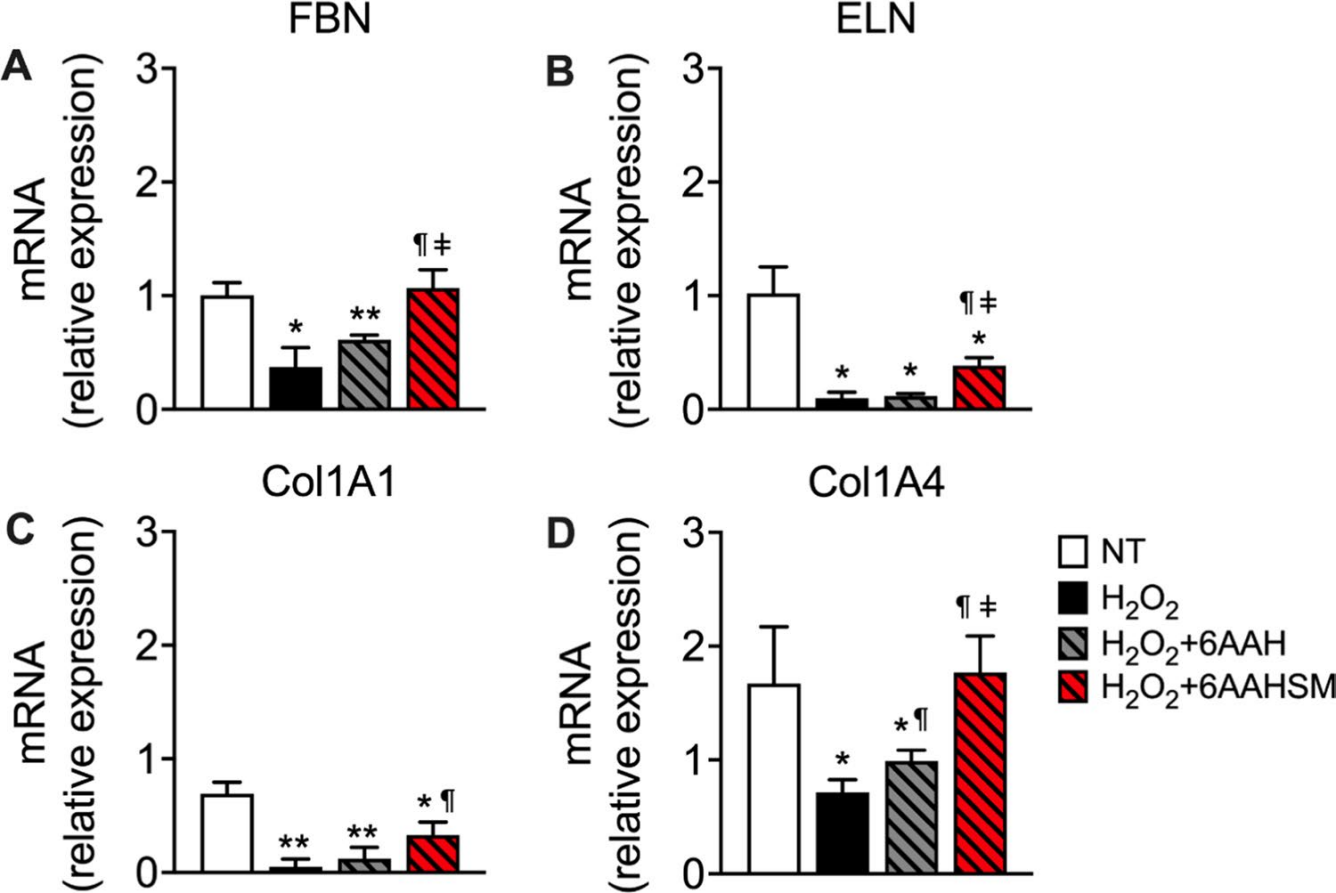
6AAHSM protects ECM from oxidative stress. RT-PCR analysis of ECM markers in hydrogen peroxide (H_2_O_2_) treated BJ fibroblasts. NT: untreated controls, H_2_O_2_: hydrogen peroxide, 6AAH: six-aminoacid mixture plus hyaluronic acid, 6AAHSM six-aminoacid mixture plus hyaluronic acid, succinate, and malate. Data are Mean ± SEM of triplicate samples of two experimental replicates. ^*^P < 0.05 and ^**^P < 0.01, vs. NT, ^¶^P < 0.05 vs. H_2_O_2_, ^ǂ^*P* < 0.05 vs. 6AAH + H_2_O_2_. One-way ANOVA followed by Tukey’s post hoc test

Having established that 6AAHSM was the most effective combination in inducing the expression of ECM genes, we next set out to investigate if this ability would also result in a superior capacity to protect human fibroblasts from an oxidative challenge with respect to 6AAH mixture alone. To this end, BJ cells were pre-treated with hydrogen peroxide (H_2_O_2_) or H_2_O_2_ plus 6AAH, or H_2_O_2_ plus 6AAHSM, and ECM gene expression was assessed. As expected, while H_2_O_2_ treatment downregulated the expression of all ECM genes, 6AAH was ineffective in restoring their expression, except for Col1A4, which was 6AAH significantly increased when compared to H_2_O_2_ alone (Fig. 3). However, 6AAHSM treatment in H_2_O_2_ fibroblasts significantly reinstated the peroxide-induced decrease of all ECM genes, with FBN and Col1A4 expression completely restored to control levels.

These data suggest that adding TCA cycle intermediates to the 6AAH mixture enhances its antioxidant capacity.

## Discussion

This paper shows that a 6AA mixture plus HA (6AAH) added with TCA cycle intermediates significantly stimulates ECM gene expression in human fibroblasts. In addition, combining succinate plus malate has an additive effect compared to malate or succinate alone. We have previously shown that the 6AA mixture upregulated the expression of ECM genes. However, in that previous work, 6AA mixture was effective only at a final concentration of 1%, while 0.1% was ineffective in inducing ECM expression genes (Tedesco et al. 2022). In the present manuscript, we show that, when combined with TCA cycle intermediates, with a more pronounced effect when succinate and malate are added contemporarily, the AA mixture is able to increase ECM expression genes even at the lower 0.1% concentration. The function of AAs as anaplerotic substrates and oxidative fuels of the mitochondrial TCA cycle is well known (Owen et al. 2002); as such, AA supplementation has been shown to powerfully stimulate mitochondrial biogenesis, which, in turn, results in positive outcomes on several age-associated and metabolic diseases (Nisoli et al. 2008; D’Antona et al. 2010; Tedesco et al. 2020). Little is known about mitochondria’ role and function and/or other metabolic pathways in ECM deposition (Romani et al. 2021). However, given the high energy requirements for its synthesis, an increase in mitochondrial function should positively affect matrix deposition. Our data seem to confirm this hypothesis: although in the current work, we did not measure mitochondrial biogenic markers following treatment with our mixtures, several experimental pieces of evidence show that TCA cycle substrates stimulates mitochondrial function. In particular, pyruvate increases mitochondrial biogenesis in vitro, and a mixture of pyruvate, malate, and citrate was found to enhance mitochondrial respiration in myotubes, while malate supplementation increased aerobic capacity in rats (Wilson et al. 2007; Wu et al. 2007; Merante 2020).

Most importantly, we have previously demonstrated that an AA mixture enriched with branched-chain amino acids plus succinate and malate (α5) efficiently boosted mitochondrial biogenesis and prevented doxorubicin-induced oxidative stress in both cardiomyocytes and mouse ventricles (Tedesco et al. 2020). In the present work, expression of all the major ECM constituents was moreover maximally increased when 6AAH mixture was added with succinate plus malate, thus indicating a positive correlation between the treatment with TCA cycle intermediates and ECM gene expression. Interestingly, a recent report using single-cell RNAseq analysis showed that in mouse cartilage, a decrease in expression of mitochondrial DNA-encoded electron transport chain genes is associated with alterations in ECM integrity (Bubb et al. 2021), supporting our hypothesis that increasing mitochondrial biogenesis would lead to a stimulation of matrix deposition.

Some hypotheses can be made regarding the mechanism of action underlying the ability of the 6AAHSM to boost ECM expression above than the other formulations: the AA composition of our mixture resembles that of the functional domains of Eln and Col, which are enriched in glycine, valine, proline, and alanine, and glycine and proline, respectively, while glycine, leucine, and valine are also abundant in Fbn primary sequence. Therefore, one possibility could be that the delivery of AA substrates could stimulate the synthesis of ECM proteins. In this regard, the co-treatment with succinate plus malate could also provide the mitochondrial ATP output for protein synthesis, which, in turn, would further stimulate the transcription of the other ECM genes. In line with this, 6AAHSM significantly increases Fbn protein levels (Fig. 2); alternatively, TCA cycle intermediates could directly, or through the reprogramming of metabolic pathways, influence the activity of the transcription factor machinery underlying ECM expression. Further studies are needed to clarify this point.

Notably, we also show that 6AAHSM can restore the oxidative stress-induced downregulation of ECM gene expression. Since excessive ROS exposure is one of the primary causes of skin aging, our finding is a relevant result; in fact, it implies that the anti-ROS function of TCA cycle intermediates could also be employed in formulations aimed to counteract the aging of the skin, similar to those we demonstrated able to block doxorubicin-induced oxidative stress in mouse hearts (Tedesco et al. 2020). Of note, contrarily to the data on ECM gene expression, 6AAH alone was also somewhat effective, albeit not significantly, in blunting the decrease in ECM gene expression resulting from H_2_O_2_ treatment, thus indicating a major role for our AA formulation in protection from oxidative damage, which was further potentiated by TCA cycle intermediates. However, since AA-defined mixtures increase mitochondrial biogenesis, we cannot exclude a mitochondrial-mediated anti-ROS activity also in the effects of the 6AAH mixture alone.

In conclusion, here, we have shown that treatment of human fibroblasts with a specific AA mixture enriched with succinate plus malate stimulates ECM deposition and protects ECM from oxidative damage. Our results suggest that the combination of specific AAs with TCA cycle intermediates could be a valuable non-pharmacological approach, paving the way for new formulations to combat skin aging. Furthermore, our mixture could be also a useful tool for investigating other cellular processes such as the mechanism governing ECM deposition or protection from oxidative stress.

## Data Availability

The data underlying this article are available in the article.
